# A Low-Cost Tele-Imaging Platform for Developing Countries

**DOI:** 10.3389/fpubh.2014.00135

**Published:** 2014-09-05

**Authors:** Kokou Adambounou, Victor Adjenou, Alex P. Salam, Fabien Farin, Koffi Gilbert N’Dakena, Messanvi Gbeassor, Philippe Arbeille

**Affiliations:** ^1^Unité de Télémédecine du Centre Hospitalier Universitaire Campus, Université de Lomé, Lomé, Togo; ^2^Unité de Médecine et Physiologie Spatiales (UMPS-CERCOM), Centre Hospitalier Universitaire Trousseau de Tours, Tours, France; ^3^Chelsea and Westminster Hopital, London, UK

**Keywords:** tele-imaging, tele-expertise, tele-diagnosis, low-cost, developing country

## Abstract

**Purpose:** To design a “low-cost” tele-imaging method allowing real-time tele-ultrasound expertise, delayed tele-ultrasound diagnosis, and tele-radiology between remote peripherals hospitals and clinics (patient centers) and university hospital centers (expert center).

**Materials and methods:** A system of communication via internet (IP camera and remote access software) enabling transfer of ultrasound videos and images between two centers allows a real-time tele-radiology expertise in the presence of a junior sonographer or radiologist at the patient center. In the absence of a sonographer or radiologist at the patient center, a 3D reconstruction program allows a delayed tele-ultrasound diagnosis with images acquired by a lay operator (e.g., midwife, nurse, technician). The system was tested both with high and low bandwidth. The system can further accommodate non-ultrasound tele-radiology (conventional radiography, mammography, and computer tomography for example). The system was tested on 50 patients between CHR Tsevie in Togo (40 km from Lomé-Togo and 4500 km from Tours-France) and CHU Campus at Lomé and CHU Trousseau in Tours.

**Results:** A real-time tele-expertise was successfully performed with a delay of approximately 1.5 s with an internet bandwidth of around 1 Mbps (IP Camera) and 512 kbps (remote access software). A delayed tele-ultrasound diagnosis was also performed with satisfactory results. The transmission of radiological images from the patient center to the expert center was of adequate quality. Delayed tele-ultrasound and tele-radiology was possible even in the presence of a low-bandwidth internet connection.

**Conclusion:** This tele-imaging method, requiring nothing by readily available and inexpensive technology and equipment, offers a major opportunity for telemedicine in developing countries.

## Introduction

Tele-radiology is the practice of radiology at a distance via the use of emerging information and communication technologies (ICTs). It consists of producing a radiological image [X-ray (XR), ultrasound (USS), magnetic imaging resonance (MRI), nuclear medicine (NM)] and making said image available to an off-site radiologist through the use of telecommunications systems for the purpose of obtaining radiological expertise that is unavailable on-site.

Dr. Kenneth Bird first conceived of tele-radiology in the late 1960s, when he used a televisual transmission system based on radio waves between the Massachusetts General Hospital (USA) and Boston’s Logan airport (USA), at a distance of 5 km (1). However, Andrus was the first to formerly publish a report on the use of tele-radiology, when he successfully completed in 1972 the transmission and interpretation of images using radio waves between two sites 50 km apart (1). Since then, tele-radiology has benefited enormously from technological progress in the fields of information technology and telecommunications, and as a result is now one of the most developed and practiced forms of tele-medicine (2–4).

The main objective of this technology is the exchange and sharing of medical images between health professionals for the purpose of obtaining a diagnosis at a distance, either in real time or following a time delay. It is perhaps one of the best practical solutions to a shortage of on-site experienced radiologists, particularly in developing countries. In the developing world in particular, the presence of radiologists is often fragmented within a country. Tele-radiology therefore has the potential to play a key role in medicine in bridging the gap in equal access to diagnostic imaging.

Togo, a country with a population of six million inhabitants as of 2012, has approximately 10 radiologists, of which 90% are based at Lomé, the capital. As a result, ultrasound, a non-irradiating imaging modality, which is readily available throughout developed countries and is often used in emergency situations due to its diagnostic utility, portability, and ease of use, is rarely available in rural areas. In many developing countries, the unavailability of ultrasound is often due to both a consequence of inadequate human resources (i.e., sufficient radiologists or sonographers), as well as a lack of equipment due to, historically at least, the high costs involved in purchasing and maintaining the equipment. Recently, however, ultrasound equipment has become more affordable with the advent of low-cost Asian manufacturers (5). However, even if a radiologist or sonographer is present and available on-site, they may not be competent in all aspects of ultrasound technique. For these reasons, patients who present to rural and remote clinics with medical conditions that require radiological investigation, are often transferred to urban centers, even when their medical condition would be best managed on-site. This is often a massive burden on patients who have financial limitations and are unable to cover the costs of transport and social care.

Access to high bandwidth internet connection and the high cost of tele-radiology technology and equipment has historically represented a barrier to the use and spread of tele-radiology in developing countries, where the need is often greatest. The purpose of this study therefore was to develop an integrated tele-ultrasound, tele-radiology, and tele-information method for use between experts at a tertiary medical center (CHU) and remote medical facilities (peripheral hospitals and rural clinics), of minimal cost and possible with relatively low-bandwidth internet connections, that would allow its implementation in low-income countries such as our own.

## Materials and Methods

### Equipment and technology for data transmission in the presence of high bandwidth: Axis 207W network camera and Axis 243SA video server

A network camera (Axis 207W – address IP) is installed in the remote center (patient center) and the expert center. These two cameras allow for both audio and video transmission between computers at the patient center and the expert center via an internet connection. This therefore enables videoconferencing between the two centers. An internet video server (Axis 243SA – address IP) is connected to an ultrasound machine (any commercial machine) at the patient center and allows for the real-time transmission of ultrasound video sequences via the internet to the computer at the expert center. The Axis 207W network camera et Axis 243SA video server benefit from a full package of security functions, including multiple user access levels with password protection, HTTPS encryption, IP address filtering, thus ensuring secure video handling and configuration.

### Equipment and technology for data transmission in the presence of low bandwidth: LogMeIn software

LogMeIn is a remote access software. It enables one to connect to a host computer from another computer or device (client) at any time, as long as an internet connection is available. Two LogMeIn products exist: LogMeIn Free and LogMeIn Pro. LogMeIn Free is free online and LogMeIn Pro requires a subscription. Following installation of the LogMeIn Pro software on the host computer, it is possible to access the software from any internet enabled computer or mobile device (LogMeIn for iOS or LogMeIn Ignition for Android).

LogMeIn Pro has a multitude of functions, including transmission of high definition quality video, audio transmission, file transfer, file and desktop sharing, control of multiple monitors, password saving, alerts with LogMeIn central, remote awakening from standby, and system diagnostics.

The computers from which one is running and remotely accessing LogMeIn must meet the following system requirement:

#### Host system requirement

- Windows 7, Vista, XP, Server 2003, 2008 (64 bits)
- Windows ME and 2000 (32 bits)
- Mac OS 10.4 (Tiger), 10.5 (Leopard), 10.6 (Snow Leopard), and 10.7 (Lion) on Mac computers equipped either with a Power PC or Intel processor.

#### Client system requirements

- Internet Explorer (IE) 6 or a later version (128 or 256 bits). IE7 or later is recommended.
- Firefox 3.6 or later.
- Google Chrome 2.0 or later.
- Safari 4.1 or later (Mac only).
- To use a tablet device or smartphone as client, LogMeIn for iOS or LogMeIn Ignition for Android.

The LogMeIn software, by allowing a radiologist at the expert center to take control of the computer at the patient center, via laptop for example, renders possible not only still image transfer (XR, USS, CT, MRI, NM), but also tele-ultrasound expertise in the presence of an inexperienced sonographer or untrained user at the patient center. In the absence of a sonographer at the patient center, an expert is able to make a radiological diagnosis in delayed time from ultrasound video sequences sent by an untrained user, with the use of the 3D virtual navigation program ECHO-CNES (Unité de Médecine et Physiologie Spatiales de Tours) (6). For tele-ultrasound, the computer at the patient center (isolated site) is beforehand connected to the ultrasound device via a video converter/USB (Pinnacle for example). Almost all the ultrasound devices have a port (bearing) for video converters.

LogMeIn includes a security system not only based on a 256 bit SSL/TLS encryption, but also multiple authentication systems. In effect, LogMeIn has many systems of authentication, including authentication of the gateway to the Client, authentication of users to the gateway, authentication of the gateway to the host, and authentication of the host to the gateway, ensuring the security of the computer data at both the patient and expert center.

Finally, LogMeIn can be coupled with Skype to enable audio and face-to-face communication between the operators at the two centers. Skype is a free software available online that includes a call notification system and contacts lists.

### Pilot experiment for the tele-radiology platform

For this pilot study, the remote patient center was CHR Tsévié situated 35 km from Lomé, capital of Togo. CHR Tsévié has a general medicine service, an internal medicine service, and a radiology service that includes a 2D ultrasound machine (GE Logiq 200) but no on-site radiologist. The expert centers were CHU at Lomé and CHU Trousseau de Tours in France (approximately 4500 km from Tsévié). An internet connection was installed at CHR Tsévie (fiber-optic) and at CHU Lomé (ADSL) for the study. The transmission speed of the internet connection had been measured by online free software such as Speed Test. The approximate averages of the frame rate and delay transmission of the ultrasound video sequences determined when the actual speed measured is very close to that theoretical claimed by the Internet Service Provider were considered. The CHU at Lomé is the second biggest tertiary referral center in Togo. The system was tested with 50 patients at CHR Tsévie. Patients gave full informed consent. These patients were either recruited upon emergency admission to hospital or were already hospitalized at CHR Tsévie. Diagnostic imaging requests were ordered initially by the doctors at CHR Tsévie or following a telemedicine consultation. The experts were university hospital radiologists. The imaging requests were ordered predominantly by general medicine doctors at CHR Tsévie.

The quality of the images tele-transmitted were appreciated by three expert radiologists (University hospital radiologist), the appreciation retained for the quality of the transmitted images for every bandwidth was that of at least two of the three expert radiologists.

## Results

A bandwidth of a minimum of 1 Mbps was necessary for the transmission of real-time ultrasound video sequences, but also background video from the remote center with the Axis technology. The quality of the ultrasound images tele-transmitted by the video server Axis 243SA was sufficient (minimum frame rate about 10 fps) for an accurate diagnosis with a transmission delay of approximately 1.5 s. The optimal quality of transmission (approximate average frame rate of 35 fps) was obtained with a bandwidth of 4 Mbps, which transmission delay was about 0.5 s.

With an average bandwidth of 512 kbps, LogMeIn allowed us to transmit images and video sequences of satisfactory quality with a delay of 2 s. With a bandwidth of 256 kbps, we noticed with LogMeIn a distortion of color ultrasound video sequences, although static images were of acceptable quality. LogMeIn was also tested with 3G dongle internet connections (SFR, Helim de TogoTelecom). Video sequences and static images were of satisfactory quality using this.

The LogMeIn file transfer function permitted the direct transfer of video files saved at the patient center computer to the computer at the expert site, enabling delayed post-treatment with the ECHO-CNES navigation program. A file of 100 Mb in size took about 3 min to transfer.

**Figure 1:**
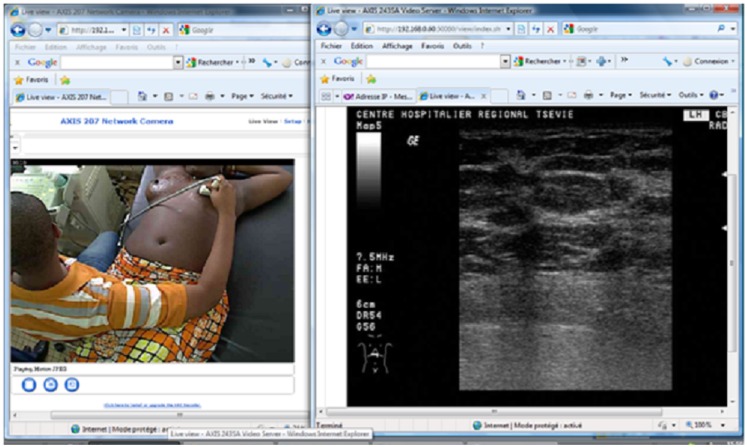
**Screen capture at the expert center during mammary ultrasound with Axis technology**.

**Figure 2:**
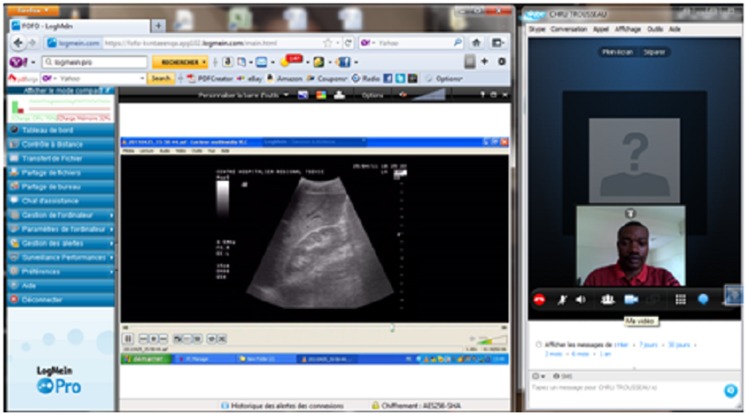
**Screen capture at the expert center during an abdominal ultrasound with LogMeIn**.

**Figure 3:**
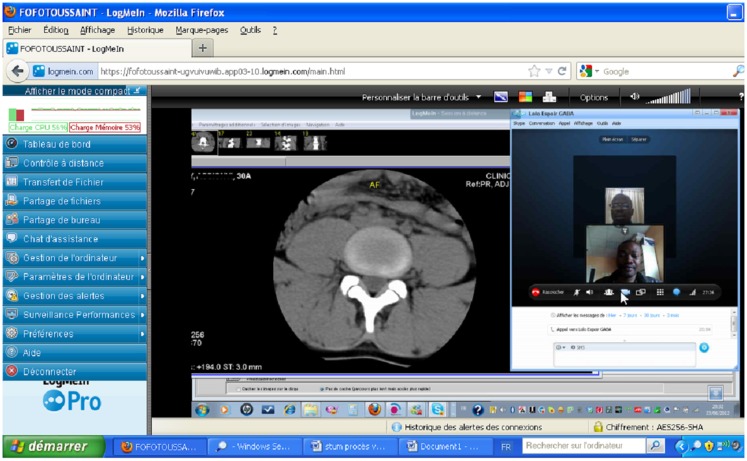
**Tele-radiology screen capture (vertebral CT scan interpreted remotely using LogMeIn)**.

The use of Axis technology with an average internet connection of 2 Mbps enabled the experts at the CHU campus and the CHU Trousseau de Tours en France to perform highly satisfactory real-time tele-ultrasound consultations for 28 ultrasounds: abdominal (*n* = 10), pelvic (*n* = 6), obstetric (*n* = 4), prostate (*n* = 4), and breast (*n* = 4) (Figure 1). The coupling of LogMeIn with Skype with a bandwidth of 512 kbps allowed for both tele-ultrasound and videoconferencing capabilities in 15 cases (Figure 2). For these tele-ultrasound videoconferences, the ultrasound operator at the CHR Tsévie was either a radiology intern sent to the CHU specifically for the case or the gynecologist at CHR Tsévie. We were also able to perform seven cases of tele-radiology using LogMeIn relating to conventional XR, computer tomography, and mammograms (Figure 3).

With inexperienced ultrasound operators at CHR Tsévie (e.g., radio operators, nurses, midwives), 10 delayed-time diagnostic tele-ultrasound cases were performed with the virtual navigation program ECHO-CNES. These tele-ultrasound sessions enabled a degree of gradual training at a distance of non-experts (e.g., midwives).

## Discussion

The Axis 207W camera server and the Axis 243SA video internet server are readily commercially available. The Axis 207W camera server was originally designed and intended for indoor video surveillance and to be able to be controlled remotely. The Axis 243SA video internet server is used in any facility where an analog surveillance system is already installed but the passage in the digital technology is imperative. This is the first time Axis video internet servers and technology have been used in medical tele-radiology and represents an innovative use.

The Axis 243 video server costs only 850 euros and the Axis 207 video camera costs only 350 euros. 1500 Euros is therefore enough using this technology to implement a tele-radiology platform between a remote center and a center of expertise. The monthly cost of internet connection in Togo actually for 512 kbps is about 46 euros, for 1 Mbps is 89 euros, and for 2 Mbps is 172 euros. These costs, which to date are one of the most expensive in West Africans countries, will certainly be reduced in the next coming years in the wake of the new policy of telecommunication committed by the government. We have previously used Axis technology in conjunction with a cloud based file hosting service (Dropbox) as a low-cost platform for obstetric and gynecological tele-imaging (7). The preliminary technical and clinical results have also been published (8).

The Axis technological infrastructure requires a minimum bandwidth of 1 Mbps for a transmission of acceptable quality. While this level of bandwidth is relatively small when compared with the real-time cardiological tele-consultation and tele-echocardiography platform proposed by Boman, which requires a bandwidth of 20 Mbps (9), such a bandwidth will not necessarily be available in rural zones in developing countries (10). One of our aims therefore was to conceive of a system based around the LogMeIn software, which is not only affordable, but also functional in areas with low-bandwidth internet, such as developing countries like Togo.

LogMeIn is available online and the basic version is free. The pro-version costs only 53 euros per year per computer. Other than the low cost, the high level of security available with LogMeIn compared with many other remote access programs was another factor that attracted us to this piece of software. LogMeIn has two intrusion detection capabilities: SSL/TLS et the LogMeIn intrusion filters. The first level of intrusion detection is based around SSL/TLS in order to detect the possible modification of data in transit. The second layer of security relies on three intrusion filters, namely an IP address filter, a denial of service filter, and an authentication filter.

The file transfer capability available with the LogMeIn Proversion, which allowed transfer of ultrasound files up to 100 Mb in around 3 min, demonstrates clearly that this software can be used for delayed tele-ultrasound diagnosis with ECHO-CNES in the presence of a non-expert at the patient center.

The LogMeIn technological infrastructure, which functioned perfectly well with 3G dongles and modest bandwidth offers therefore a practical mechanism for medical tele-imaging services even in the most remote areas in which information and communications technologies are lacking. Further, tele-ultrasound expertise could be made available for patients in ambulances with the use of the LogMeIn software. All that is required is a portable ultrasound on board the ambulance and a 3G dongle for internet connection. One can also envisage the expert being able to view the images on a tablet device or smartphone with the use of LogMeIn for iOS or LogMeIn for android.

With untrained ultrasound operators at CHR Tsévié, only delayed tele-ultrasound diagnosis with the virtual navigation program ECHO-CNES was possible. This 3D reconstruction program, of which the diagnostic accuracy for general abdomen ultrasound was estimated at 91% in a previous study (6), enables the expert to review the images sent by the untrained person at a later time and make a radiological diagnosis. Ultrasonography involves a degree of technical skill and precision, and thus it is quite operator dependent. As a result, it can be difficult to guide a lay person remotely by voice and/or video. The difficulty in realizing real-time tele-ultrasound with an untrained operator has led some authors to produce visual guides to assist in obtaining images of the organ of interest for diagnosis (11, 12). Sheehan et al., for example, have produced a guide (Expert Visual Guidance) (11) consisting of a program that shows the various positions and angles of the ultrasound probe necessary to obtain the required views of the organ of interest. The authors tested the program with 20 medical students with no prior ultrasound experience. Compared to a group of medical students who were guided by voice alone, these untrained students were more competent at obtaining the necessary views for making a diagnosis.

Other authors have used robotic systems, ESTELE (13), OTELO (14), TER (15), to allow an expert to remotely manipulate the ultrasound probe on the patient. These remote-control systems include a robotic arm at the end of which is fixed an ultrasound probe, which an untrained person places and maintains on the patient. The radiologist at the expert center holds a fictitious probe, and via this it is able to tele-manipulate the ultrasound and obtain different ultrasound views in real time. The robotic arm at the patient center reproduces with high fidelity the hand movements of the radiologist at the expert center. The high cost of such robotic systems, however, renders them unaffordable in low-income countries such as Togo.

The CHU Trousseau de Tours in France was one of the expert centers in this study. The ability to have specialists make a diagnosis at a distance from France thanks to our method would significantly improve the care of certain patients and avoid the need for costly repatriations to European centers in some cases. The beneficial role that such a platform can play was apparent when the “télésanté en Afrique” project was piloted by Rovetta et al. in 1997, in which doctors in France and Italy performed successful tele-medicine consults with hospitals in the Ivory coast and South Africa (16). Further, Gimel et al. (17) demonstrated the feasibility of a tele-pathology service between dermo-pathologists at the Massachusetts General Hospital in the United States and African hospitals, via the transmission of static histology images. The availability and combination of free, yet secure, software such as LogMeIn and low-cost equipment such as Axis technology and their use in developing countries, as used in this study, will hopefully further stimulate the introduction of tele-medicine networks between reference centers in developed countries and remote centers in developing countries.

The fact that both patient and medical personnel adherence was good in this study is highly encouraging. Several other telemedicine projects used throughout the world, although innovative, have ultimately failed due to a lack of interest by users (18).

We have not as yet carried out a study in which we had sufficient *n* numbers to achieve statistical significance with Cohen’s Kappa (a limitation of this current study). Nevertheless, our work appears reproducible. However, we organized a workshop during the ninth Société de Radiologie de l’Afrique Noire Francophone (SRANF) in May 2011 in Lomé in which we successfully demonstrated, in particular, tele-ultrasound using our system. Numerous eminent African radiologists carried out tele-ultrasound sessions from the congress hotel with patients at remote centers and were satisfied that the quality of the ultrasound images was sufficient for an accurate diagnosis.

The preliminary results from this study suggest that the functionality of our low-cost tele-radiology method is satisfactory. Nonetheless, future studies using larger sample sizes will be required for clinical validation and to determine the feasibility of our method on larger scales.

## Conclusion

In this pilot study, we configured a system for medical tele-imaging using hardware that was low cost and online software that was free, demonstrating its feasibility in low-income countries. The results were highly satisfactory and offer encouraging potential for the development of telemedicine in developing countries. Such countries often suffer from a chronic shortage of medical specialists and their means are often limited in comparison to their huge healthcare needs. Despite its low cost and ease of use, the political will needs to exist to enable the large scale and systematic roll out of a method such as ours in remote areas in developing African countries.

## Conflict of Interest Statement

The authors declare that the research was conducted in the absence of any commercial or financial relationships that could be construed as a potential conflict of interest.
